# Multicenter evaluation of the QIAstat-Dx Gastrointestinal Panel 2, a multiplex PCR platform for the diagnosis of acute gastroenteritis

**DOI:** 10.1128/jcm.01983-24

**Published:** 2025-07-11

**Authors:** Wendy A. Szymczak, Anne Line Engsbro, Jan Gorm Lisby, Juan José González-López, Paul Granato, Nathan Ledeboer, Donna M. Wolk, Stephen Young, Daniel D. Rhoads, Yvan Caspar, Lisa Steed, Romney Humphries, Christopher Bielefeldt, Markus Hermanowski, Juana L. de Diego, Hendrik Leibhan, Pau Boher, Carla Camprubí, Maria Orthodoxou, Ester Sala, Sarah Johnson, Martí Juanola-Falgarona, Davide Manissero, Johanna Bialas

**Affiliations:** 1 Department of Pathology, Albert Einstein College of Medicine and Montefiore Medical Center 2006, Bronx, New York, USA; 2 Copenhagen University Hospital and Hvidovre 53167, Copenhagen, Denmark; 3 Department of Clinical Microbiology, Hospital Universitari Vall d'Hebron 38176, Barcelona, Spain; 4 CIBER de Enfermedades Infecciosas (CIBERINFEC), Instituto de Salud Carlos III 38176, Madrid, Community of Madrid, Spain; 5 Department of Genetics and Microbiology, Universitat Autònoma de Barcelona 196650, Bellaterra, Spain; 6 Laboratory Alliance of Central, Liverpool, New York, USA; 7 Department of Pathology, Medical College of Wisconsin 5506, Milwaukee, Wisconsin, USA; 8 Geisinger Diagnostic Medicine Institute, Danville, Pennsylvania, USA; 9 Geisinger Commonwealth School of Medicine 150362 , Scranton, Pennsylvania, USA; 10 TriCore Reference Laboratories 159777 , Albuquerque, New Mexico, USA; 11 Department of Pathology and Laboratory Medicine, Cleveland Clinic 685191 , Cleveland, Ohio, USA; 12 Department of Pathology, Cleveland Clinic Lerner College of Medicine, Case Western University School of Medicine 161821, Cleveland, Ohio, USA; 13 Infection Biology Program, Lerner Research Institute, Cleveland, Ohio, USA; 14 Laboratoire de Bacteriologie - Hygiene Hospitaliere, CHU Grenoble Alpes 36724 , La Tronche, Auvergne-Rhône-Alpes, France; 15 Université Grenoble Alpes, CEA, CNRS, IBS, Grenoble, France; 16 Medical University of South Carolina 2345, Charleston, South Carolina, USA; 17 Vanderbilt University Medical Center 12328, Nashville, Tennessee, USA; 18 Christus Health 275410 , Beaumont, Texas, USA; 19 Labor Berlin - Charite Vivantes GmbH 646268 , Berlin, Germany; 20 STAT-Dx Life, S.L. (a QIAGEN Company), Baldiri Reixac, Barcelona, Spain; 21 QIAGEN Manchester Ltd, Citylabs 2.0 118818 , Manchester, England, United Kingdom; National Institute of Allergy and Infectious Diseases, Bethesda, Maryland, USA

**Keywords:** gastrointestinal infection, diarrhea, multiplex PCR, syndromic testing, gastrointestinal panel, QIAstat-Dx

## Abstract

The QIAstat-Dx Gastrointestinal Panel 2 (GI2 Panel) is a sample-to-answer multiplex PCR instrument that can detect 17 targets in a run time of about 80 minutes. The performance of the QIAstat-Dx GI2 Panel was evaluated by testing 1,939 prospective, 119 prospectively collected and then archived positive clinical samples and 750 retrospective clinical specimens across 13 sites in Europe and the United States. Specimens tested included bulk stool samples preserved in modified Cary-Blair transport medium. For most targets, results were compared to those of the FilmArray GI panel (13/17), and discordant results were adjudicated with a third assay. For the remaining targets (4/17), a composite comparator method was used, which included three comparator assays for each target. Before discordant resolution, the QIAstat-Dx GI2 Panel positive percent agreement (PPA) was 95% or greater for 5/17 targets (*Campylobacter*, *E. coli* O157, *Cryptosporidium, Cyclospora cayetanensis,* and *Giardia lamblia*) and 90% or greater for 11/17 targets: adenovirus F40/F41, astrovirus, norovirus GI/GII, rotavirus A, *Plesiomonas shigelloides*, enteropathogenic *Escherichia coli*, enterotoxigenic *E. coli*, *Salmonella*, *Yersinia enterocolitica,* Shiga-like toxin *E. coli* (STEC) *stx1/stx2*, and *Shigella*/enteroinvasive *E. coli*. No cases of *Entamoeba histolytica* were encountered during the clinical study. The negative percent agreement (NPA) was >98.9% for all QIAstat-Dx GI2 Panel targets. The three most common pathogens identified in single and co-infections were enteropathogenic *E. coli* (9.9%), *Campylobacter* (5.2%), and norovirus GI/GII (3.1%). In summary, this clinical study examined more than 2,800 samples from Europe and the U.S. using the QIAstat-Dx GI2 Panel and identified 90%–100% PPA and 99% NPA for its 17 targets.

**IMPORTANCE:**

The manuscript highlights the significance and impact of the QIAstat-Dx GI2 Panel, a sample-to-answer multiplex PCR instrument capable of detecting 17 targets in approximately 80 minutes. This comprehensive clinical study, conducted across 13 sites in Europe and the United States, evaluated the performance of the panel using over 2,800 clinical samples. The results demonstrate a high accuracy of the QIAstat-Dx GI2 panel, with a PPA equal to or higher than 90% for all targets and an NPA greater than 98.9% for all targets. These findings underscore the reliability and effectiveness of the GI2 panel in the rapid and precise detection of gastrointestinal pathogens, which is crucial for timely diagnosis and treatment of infections.

## INTRODUCTION

Acute gastroenteritis poses a serious threat to global public health. Diarrhea, the main symptom of acute gastroenteritis, continues to cause morbidity and mortality in communities worldwide. Approximately 2 billion cases of diarrhea affect both adults and children each year, with 1.9 million fatalities among children aged <5 years (1). Diarrhea is the world’s eighth leading cause of mortality and the fifth leading cause among children under 5, with 90% of that mortality occurring in Southern Asia and Sub-Saharan Africa (2). Although patients in high-income countries experience lower mortality rates associated with acute gastroenteritis, large numbers of cases are reported annually: an estimated 48.9 million and 65.4 million cases, respectively, in North America and Europe (2). Acute gastroenteritis also has deleterious effects on healthcare systems and economies. In the United States, infections mediated by 14 major foodborne pathogens resulted in an annual loss of an estimated $14 billion in morbidity-related costs and 61,000 quality-adjusted life years (3). In North America and Europe, acute gastroenteritis cases often result in outpatient visits and hospitalizations, further placing a burden on healthcare facilities and leading to losses in productivity (4–6). Certain populations are more vulnerable to severe disease and complications associated with acute gastroenteritis, including the elderly (>65 years old), young children (<5 years old), and the immunocompromised (7–9).

Traditionally, acute gastroenteritis was diagnosed by testing stool specimens using conventional methods (e.g., bacterial culture, microscopy, and antigen tests) that are labor-intensive, technically complex, time-consuming, and require selecting several appropriate tests to identify the pathogens that can cause indistinguishable clinical symptoms (10). Timely and accurate identification of the causative pathogen(s) can be essential for informing appropriate diagnosis and patient care (11–17). In recent decades, syndromic panel-based testing utilizing a multiplex PCR approach has revolutionized infectious disease diagnostics with the capability to detect more than one pathogen in a clinical sample within hours (18–22). A study from (12) had a nearly twofold higher detection rate when using the QIAstat-Dx Panel compared with targeted microbiological assays. Eighty-six (68.8%) out of 125 specimens were positive by QIAstat-Dx GIP versus 44 (35.2%) positive by a composite of conventional methods (12).

In this study, we evaluated the performance of the QIAstat-Dx Gastrointestinal Panel 2 (Qiagen, Hilden, Germany) by examining the panel’s analytical PPA and NPA in a multicenter, international prospective study using stool specimens in modified Cary-Blair transport medium collected via FecalSwab (Copan, Brescia, Italy) or Para-Pak C & S medium (Meridian Bioscience, Cincinnati, OH). The diagnostic accuracy study had multiple options, comparing the QIAstat-Dx Gastrointestinal Panel 2 to the following: i) the FilmArray GI panel (bioMerieux, Marcy l’Etoile, FR) alone for thirteen targets; ii) to a composite of three FDA-approved testing panels (FilmArray GI panel, Luminex xTAG Gastrointestinal Panel (Diasorin, Austin, TX) and BD MAX Enteric Panels (Becton-Dickenson, Sparks, MD)) for three targets; or iii) to a composite of two FDA-approved testing panels (FilmArray GI panel and Luminex xTAG Gastrointestinal Panel) and two validated PCR tests followed by bi-directional sequencing (BDS) for one target.

## MATERIALS AND METHODS

### Clinical specimen enrollment and collection

Enrollment of specimens and testing were carried out between May 2021 and July 2021 at 13 clinical sites representative of different geographical areas across five countries in Europe and North America.

### Prospective clinical specimens

Stool samples were prospectively collected from patients presenting signs and/or symptoms of gastrointestinal infection. These specimens were collected either using FecalSwab (Copan) or Para-Pak C & S medium (Meridian) according to the manufacturers’ instructions (Tables 1 and 2). Briefly, for FecalSwab, the flocked swab was inserted, rotated into the stool sample, and transferred into the tube medium not exceeding the “fill line.” For Para-Pak C&S, the provided sampling paddle was used to collect enough stool to reach the “fill line” indicated on the tube. Both collection and transportation media are based on the modified Cary-Blair medium, which utilize a non-nutritive, buffered solution to ensure survival of enteric pathogenic bacteria and prevent overgrowth by commensal organisms.

**TABLE 1:** Institutions that tested clinical prospective specimens in this investigation

Site code	Country (state)	Prospective clinical specimens analyzed	
		FecalSwab	Para-Pak C&S
US-01	USA (New York)	0	186
US-02	USA (South Carolina)	0	43
US-03	USA (New York)	282	0
US-04	USA (Wisconsin)	0	177
US-05	USA (Tennessee)	44	0
US-06	USA (Louisiana)	0	39
US-07	USA (Pennsylvania)	0^*a*^	0^*a*^
US-08	USA (New Mexico)	0	131
US-10	USA (Ohio)	0	95
DE-01	Germany	293	46
DK-01	Denmark	293	0
FR-01	France	63	0
ES-01	Spain	247	0

^*a*^ The specimens from this site were excluded from the analysis because they were collected with another device different from Para-Pak C&S or FecalSwab.

**TABLE 2:** Summary of the number and percentage of positive results by age group and sample type for the prospective clinical study, as determined by the QIAstat-Dx Gastrointestinal Panel 2

Pathogen	Sample type	Overall	0–5 years	6–21 years	22–49 years	50 + years
Adenovirus F40/F41	FecalSwab	5 (0.4%)	3 (1.7%)	2 (1.7%)	0 (0.0%)	0 (0.0%)
	Para-Pak C&S	2 (0.3%)	1 (3.2%)	0 (0.0%)	0 (0.0%)	1 (0.2%)
Astrovirus	FecalSwab	3 (0.2%)	3 (1.6%)	0 (0.0%)	0 (0.0%)	0 (0.0%)
	Para-Pak C&S	6 (0.8%)	2 (6.5%)	0 (0.0%)	3 (1.4%)	1 (0.2%)
*Campylobacter*	FecalSwab	69 (5.6%)	25 (13.7%)	7 (5.8%)	17 (5.9%)	20 (3.2%)
	Para-Pak C&S	30 (4.2%)	2 (6.5%)	0 (0.0%)	10 (4.7%)	18 (4.3%)
*Cryptosporidium*	FecalSwab	2 (0.2%)	0 (0.0%)	1 (0.8%)	1 (0.3%)	0 (0.0%)
	Para-Pak C&S	7 (1.0%)	0 (0.0%)	1 (2.8%)	4 (1.9%)	2 (0.5%)
*Cyclospora cayetanensis*	FecalSwab	3 (0.2%)	0 (0.0%)	1 (0.8%)	2 (0.7%)	0 (0.0%)
	Para-Pak C&S	18 (2.5%)	0 (0.0%)	0 (0.0%)	6 (2.8%)	12 (2.9%)
Enteropathogenic *E. coli* (EPEC)	Para-Pak C&S	56 (7.9%)	9 (29.0%)	2 (5.6%)	18 (8.4%)	27 (6.5%)
Enterotoxigenic *E. coli* (ETEC) *lt/st*	FecalSwab	18 (1.5%)	2 (1.1%)	2 (1.7%)	11 (3.8%)	3 (0.5%)
	Para-Pak C&S	17 (2.4%)	1 (3.2%)	0 (0.0%)	7 (3.3%)	9 (2.2%)
*Giardia lamblia*	FecalSwab	15 (1.2%)	3 (1.6%)	1 (0.8%)	7 (2.4%)	4 (0.6%)
	Para-Pak C&S	1 (0.1%)	1 (3.2%)	0 (0.0%)	0 (0.0%)	0 (0.0%)
Norovirus GI/GII	FecalSwab	43 (3.5%)	22 (12.1%)	1 (0.8%)	14 (4.8%)	6 (1.0%)
	Para-Pak C&S	16 (2.3%)	3 (9.7%)	1 (2.8%)	3 (1.4%)	9 (2.2%)
*Plesiomonas shigelloides*	FecalSwab	2 (0.2%)	0 (0.0%)	0 (0.0%)	2 (0.7%)	0 (0.0%)
	Para-Pak C&S	7 (1.0%)	1 (3.2%)	0 (0.0%)	4 (1.9%)	2 (0.5%)
Rotavirus A	FecalSwab	23 (1.9%)	13 (7.1%)	2 (1.7%)	7 (2.4%)	1 (0.2%)
	Para-Pak C&S	4 (0.6%)	2 (6.5%)	0 (0.0%)	0 (0.0%)	2 (0.5%)
*Salmonella*	FecalSwab	14 (1.1%)	5 (2.7%)	4 (3.3%)	3 (1.0%)	2 (0.3%)
	Para-Pak C&S	17 (2.4%)	4 (12.9%)	0 (0.0%)	3 (1.4%)	10 (2.4%)
Shiga-like toxin *E. coli* (STEC) *stx1/stx2*	Para-Pak C&S	9 (1.3%)	0 (0.0%)	0 (0.0%)	6 (2.8%)	3 (0.7%)
Shigella/enteroinvasive *E. coli* (EIEC)	FecalSwab	10 (0.8%)	1 (0.5%)	0 (0.0%)	6 (2.1%)	3 (0.5%)
	Para-Pak C&S	3 (0.4%)	0 (0.0%)	0 (0.0%)	1 (0.5%)	2 (0.5%)
*Yersinia enterocolitica*	FecalSwab	22 (1.8%)	3 (1.6%)	2 (1.7%)	9 (3.1%)	8 (1.3%)
	Para-Pak C&S	8 (1.1%)	0 (0.0%)	0 (0.0%)	4 (1.9%)	4 (1.0%)

Samples were residual stool specimens obtained after the completion of all routine gastrointestinal testing procedures. The preserved stool specimens were homogenous suspensions, easily vortexed, and had a minimum residual volume of 1.0 mL. The preserved stool specimens were stored for a maximum of 4 days at room temperature (15°C to 25°C) or 4 days at refrigerated conditions (2°C to 8°C) before testing. For each sample collected and tested, at least one aliquot (0.5 mL) was stored at −80°C to −70°C for further testing, if required. Prospective clinical specimens were excluded from the investigation if they (i) were received in the laboratory as frozen stool; (ii) had been centrifuged; (iii) had been contaminated with urine or water; (iv) were submitted in a damaged or leaking container; (v) lacked clear subject identification or label; (vi) were stored in medium other than modified Cary-Blair; (vii) contained formalin or other fixatives; (viii) were not collected according to the manufacturer’s instructions (overfilled or under-filled stool containers); (ix) were rectal swab specimens; or (x) submitted from a patient who had been previously enrolled in the study.

Prospectively collected clinical specimens have been used for epidemiological data presentation across this manuscript.

### Prospectively collected and then archived positive clinical specimens

A supplemental study was carried out involving the testing of positive stool specimens for STEC and Norovirus GI/GII that were prospectively collected and then archived (frozen) in order to enrich the positivity for Norovirus GI/GII and STEC through the enrolment process, within a defined time range without any sample selection. These were prospectively collected samples from four different collection sites (3 US and 1 EU), where only those positive for the pathogen by a standard of care method were archived for analysis alongside negative specimens. All prospectively collected and then archived positive clinical specimens were stored at −80°C to −70°C for up to 1.5 years, before analysis.

Prospectively collected and then archived positive clinical samples have not been used for epidemiological data presentation across this manuscript.

### Retrospective clinical specimens

Retrospective testing was carried out on archived pre-selected banked stool specimens that had previously tested positive for one of the targets on the QIAstat-Dx Gastrointestinal Panel 2, according to the clinical sites or biobank’s standard of care method. Retrospective specimen testing was carried out to enrich the positivity of pathogens with anticipated low prevalence or that were less represented in a particular sample type (Para-Pak C&S or FecalSwab). Retrospective specimens were selected to contain the following pathogens: adenovirus F40/F41, astrovirus, *Campylobacter*, *Cryptosporidium*, *Cyclospora cayetanensis*, *E. coli* O157, *Entamoeba histolytica*, enteropathogenic *E. coli* (EPEC), enterotoxigenic *E. coli* (ETEC) *lt*/*st, Giardia lamblia*, norovirus GI/GII, *Plesiomonas shigelloides*, rotavirus A*, Salmonella,* Shiga-like toxin *E. coli* (STEC) *stx1*/*stx2*, *Shigella*/enteroinvasive *E. coli* (EIEC), and *Yersinia enterocolitica*.

Retrospectively collected clinical specimens were not used for epidemiological data presentation across this manuscript.

### Testing with QIAstat-Dx Gastrointestinal Panel 2

The QIAstat-Dx Gastrointestinal Panel 2 is a newly developed multiplex PCR panel-based testing platform that can identify up to 17 targets in a stool specimen. This closed testing system is easy to use—involving minimal sample manipulation—and has a rapid turnaround time of approximately 80 minutes per sample.

The detection of 17 targets (four viruses, eight bacteria plus the detection of *E. coli* O157 serogroup within the STEC, and four parasite species) was reported. The pathogen targets in the panel were adenovirus F40/F41, astrovirus, norovirus GI/GII, rotavirus A, *Campylobacter* (*C. jejuni*, *C. coli*, and *C. upsaliensis*), *Shigella*/EIEC, EPEC, ETEC*, P. shigelloides*, *Salmonella* spp., STEC *stx1*/*stx2* (including specific identification of *E. coli* O157 serogroup within STEC), *Y. enterocolitica*, *Cryptosporidium*, *C. cayetanensis*, *E. histolytica*, and *G. lamblia*. Multiplex PCR tests were conducted as per the manufacturer’s instructions (https://www.qiagen.com/us/products/diagnostics-and-clinical-research/infectious-disease/qiastat-dx-syndromic-testing/qiastat-dx-na?catno=691421). A volume of 0.2 mL of preserved stool in modified Cary-Blair was homogenized and transferred using the transfer pipette provided with the kit into a QIAstat-Dx Gastrointestinal Panel 2 Cartridge via the cartridge’s main port and placed into a QIAstat-Dx Analyzer 1.0 within 90 minutes to initiate the testing. The QIAstat-Dx Analyzer 1.0 is an automated, closed system that extracts, amplifies, and detects nucleic acids in the sample. Within the cartridge, the sample was pretreated to enhance stool inhibitory substance removal and then homogenized. Cells were then lysed through chemical lysis and mechanical disruption with silica beads. After nuclease inactivation, nucleic acids were isolated via binding to a silica membrane in the presence of alcohol and chaotropic salts in the purification chamber. Following the elution of the purified nucleic acids from the membrane, the nucleic acids were mixed with PCR reagents and then transferred to PCR chambers containing assay probes and primers. Amplification of DNA via PCR and RNA through RT-PCR was then carried out. Real-time fluorescence was measured using TaqMan probes to generate amplification curves. The results were interpreted, and a test report was prepared.

Each QIAstat-Dx Gastrointestinal Panel 2 Cartridge contains an internal processing control. This internal control, a quantified *Schizosaccharomyces pombe* target, serves to verify that all analytical steps of the process, including homogenization, lysis of cells, nucleic acid isolation, reverse transcription, and real-time PCR, have been carried out successfully. A positive result for the internal control indicated that all the analytical steps were carried out appropriately. A negative result for the internal control meant that all negative results for the sample were invalid, but all positive results (targets detected) were still valid and kept for analyses.

During this study, testing with external controls was also conducted. Four different positive control mixes and a negative control mix were prepared and tested daily in a rotating manner such that all these control mixes were assayed in 5 days (refer to Supplemental Methods for details).

### Comparator testing

For thirteen of the seventeen targets evaluated, the FDA-approved FilmArray GI panel (bioMérieux) was used as the comparator method: adenovirus F40/F41, astrovirus, rotavirus A, *Campylobacter* spp., *P. shigelloides*, *Salmonella* spp., *Y. enterocolitica*, *Shigella* spp./EIEC, EPEC, *E. coli* O157, *Cryptosporidium* spp.*, C. cayetanensis*, and *E. histolytica*. The comparator methods for three of the seventeen targets (norovirus GI/GII, ETEC, and STEC *stx1/stx2*) were a composite of three FDA-approved test methods: the FilmArray GI panel, Luminex xTAG Gastrointestinal Pathogen Panel, and BD MAX Enteric Panels (BD MAX Enteric Viral, BD MAX Enteric Bacterial, or BD MAX Extended Enteric Bacterial Panels). For the remaining target, *Giardia duodenalis* (formerly *lamblia*), the comparator methods were a composite of two FDA-approved multiplex PCR panels (FilmArray GI panel and Luminex xTAG Gastrointestinal Pathogen Panel) and two validated PCR tests followed by BDS. The final result of the composite comparator was based on the majority decision of the outcomes of the three individual test method results.

Note that a composite comparator method was used in some cases due to data available suggesting that the performance characteristics of the FilmArray GI panel may need further support as a comparator to accurately establish the performance characteristics of the investigational device (refer to Supplemental Methods for details).

The FDA-approved assay tests were performed according to the corresponding manufacturer’s instructions (refer to Supplemental Methods for details). The PCR and bi-directional sequencing test technical information is provided below.

### Discrepancy investigation

To investigate discrepancies between the testing results of the QIAstat-Dx Gastrointestinal Panel 2 and FilmArray GI panel, three different types of assays were employed, when the remaining sample volume was available: the BD MAX multiplex PCR system (BD MAX Enteric Viral Panel, BD MAX Enteric Bacteria Panel, and BD MAX Extended Enteric Bacteria Panel) performed according to the manufacturer’s instructions, the Luminex xTAG GPP panel, or a validated single-plex PCR assay coupled with BDS of the amplified product (for adenovirus F40/41, astrovirus, rotavirus, *Campylobacter* spp., *Plesiomonas shigelloides*, *Salmonella, Yersinia enterocolitica, E. coli* O157, EPEC *Shigella/*EIEC, and *Cryptosporidium* spp.) (Table S1). Discrepant analysis was not applicable for the targets evaluated using the composite comparator approach.

### PCR followed by BDS (bi-directional sequencing)

Two PCR tests followed by bi-directional sequencing (BDS) were performed as part of a composite comparator method for *Giardia duodenalis*. The PCR assays were developed at QIAGEN. In addition, single-plex PCR followed by BDS assays was performed as tests to resolve discordances between the testing results for QIAstat-Dx Gastrointestinal Panel 2 and the comparator method, FilmArray GI panel. These assays were used to investigate discordances with the comparator test results for the following pathogens: EPEC and *Cryptosporidium* spp. The PCR assays were developed at two external biotechnology vendors, GENEWIZ, USA (Azenta Life Sciences; for detecting EPEC) and Oncocyte Inc., USA (for detecting *Cryptosporidium*). All PCR-BDS assays were validated before use (refer to Supplemental Methods for details).

#### PCR for identifying *G. duodenalis*

Nucleic acids were purified from stool samples using a QIAcube instrument, an automated nucleic acid isolation system, that can process up to 12 samples in a single batch. Following purification, 5–10 µL of template DNA was mixed with QIAGEN Multiplex PCR Mastermix (PCR buffer, HotStarTaq DNA Polymerase) and forward and reverse primers at a final concentration of 0.6 µM each. The PCRs were conducted in a C1000 Touch Thermal Cycler (BioRad) or in C1000 Touch Thermal Cycler (BioRad).

#### PCR for identifying EPEC

Extraction of genomic DNA from 200 μL to 400 µL of each fecal sample was accomplished using the QIAGEN AllPrep PowerFecal DNA/RNA Kit. A volume of 2 µL of template DNA was added to a cocktail containing KAPA2G Robust HotStart ReadyMix (PCR buffer, KAPA2G Robust DNA polymerase, MgCl_2_, and dNTPs) (MilliporeSigma), 0.5 µM forward primer, and 0.5 µM reverse primer. The resulting mixture was subjected to PCR in an Eppendorf Mastercycler Pro instrument.

#### PCR for identifying *Cryptosporidium*

Genomic DNA was purified from up to 200 mg per stool specimen using the QIAGEN AllPrep PowerFecal DNA/RNA Kit. Between 10 and 20 ng of DNA was added to a mixture containing DreamTaq Green PCR Master Mix (DreamTaq DNA Polymerase, DreamTaq Green buffer, MgCl_2_, and dNTPs), 0.5 µM forward primer, and 0.5 µM reverse primer. Amplification was performed in a LifeECO thermo-cycler instrument using PCR cycling conditions optimized for each DNA target amplified.

#### BDS (bi-directional sequencing)

In order to confirm that the DNA amplified was the intended target, BDS was carried out. The Sanger sequencing method was employed for this analysis. DNA samples were purified by using the QIAquick PCR Purification Kit (QIAGEN) using the manufacturer’s protocol. The BigDye Terminator v3.1 Cycle Sequencing Kit (Thermofisher) was utilized to set up sequencing reactions with forward and reverse primers at 5 mM together with the DNA samples in a non-skirted 96-well PCR plate (Thermo Scientific). The sequencing reactions were performed in a thermal PCR cycler (Applied Biosystem GeneAmp PCR System 9700) according to the following cycle conditions: 96°C for 10 seconds, 30 cycles of 50°C for 10 seconds, 60°C for 2.5 minutes, and 4°C on hold. The sequencing data were collected using a 3730xl DNA Analyzer Sequencer (Applied Biosystems) and were analyzed utilizing the Applied Biosystems Sequencing Analysis Software 7. Further sequencing data analysis was conducted using Geneious software v.11.0.5 or the QIAGEN Genomics Workbench. The sequences generated were also subjected to BLAST analysis on the National Center for Biotechnology Information (NCBI) database.

### Sample size

The following sample size rationale applies for each target. Assuming an underlying true PPA of 95%, there is 96.22% power to observe a PPA of at least 90.0% with a sample size of at least 50.

### Calculations and statistical analysis

The positive percentage agreement (PPA) and the negative percentage agreement (NPA) were calculated for the prospective and retrospective studies and for each sample type (Para-Pak C&S and FecalSwab) separately. To calculate the PPA and NPA of each pathogen, the number of true positive (TP), false positive (FP), true negative (TN), and false negative (FN) results were determined on the QIAstat-Dx Gastrointestinal Panel 2 relative to the comparator method(s). PPA for a pathogen was calculated as 100 x (TP/(TP +FN)). NPA was calculated as 100 x (TN/(TN +FP)). Both PPA and NPA for each pathogen were reported as a fraction and percentage together with the exact corresponding binomial two-sided 95% CI using the exact Clopper-Pearson method.

## RESULTS

For this investigation, 1,939 specimens prospectively collected from nine clinical sites in the United States and four clinical sites in Europe were evaluated (Table 1). In addition, 119 prospectively collected and then archived positive clinical samples and 750 retrospective specimens were also evaluated to supplement the results. A total of 2,808 stool specimens were collected, 1,591 and 1,217 in FecalSwab and Para-Pak C&S transport devices, respectively.

### Demographics

Within the cohort of prospective specimens, 1,070 samples (55.2%) were collected from women and 869 (44.8%) from men, and most samples came from patients aged >50 years (1,055 cases, 54.4%). Taking into account that almost 30% of the data were not available regarding the patient population, the known information showed that a total of 816 (42.1%) and 485 (25.0%) stool samples came from outpatients and hospitalized patients, respectively. The complete demographic information for the 1,939 prospective specimens is shown in Table 3. Moreover, a comprehensive graph showing the source of the pathogens according to the patients’ status within the healthcare system can be found in Fig. S2.

**TABLE 3:** Demographic data for enrolled prospective specimens analyzed in this study

Demographic data	N	%
Gender		
Female	1,070	55.2
Male	869	44.8
Age group		
0–6 years	213	11.0
6–21 years	159	8.2
22–49 years	505	26.0
50 + years	1,055	54.4
Not reported	7	0.4
Patient population		
Emergency room	75	3.9
Hospitalized	485	25.0
Immunocompromised	3	0.2
Outpatient	816	42.1
No information available	560	28.9
Number of days between symptom onset and QIAstat testing		
>7 days	89	4.6
≤7 days	162	8.3
Not reported	1,688	87.1

As shown in Fig. S1, except for norovirus GI/GII, viruses identified in fecal specimens tend to be from children and young adults. Most stool specimens with adenovirus F40/F41 detected were from patients aged ≤20 years, while most patients infected with astrovirus or rotavirus A were less than 40 years old. Unlike viruses, the bacteria species of *Campylobacter*, EPEC, *Salmonella*, and STEC *stx1/stx2* were found to be prevalent in both adults and children. In contrast, ETEC *lt/st*, *P. shigelloides*, EIEC, and *Y. enterocolitica* were mostly identified in fecal samples of adults. Similarly, most stool samples containing parasites, *Cryptosporidium* or *C. cayetanensis,* were from adults. However, it appears that *G. duodenalis*, another parasite, infected both adults and children.

### Positive fecal samples by enteric pathogen

**Fig 1:**
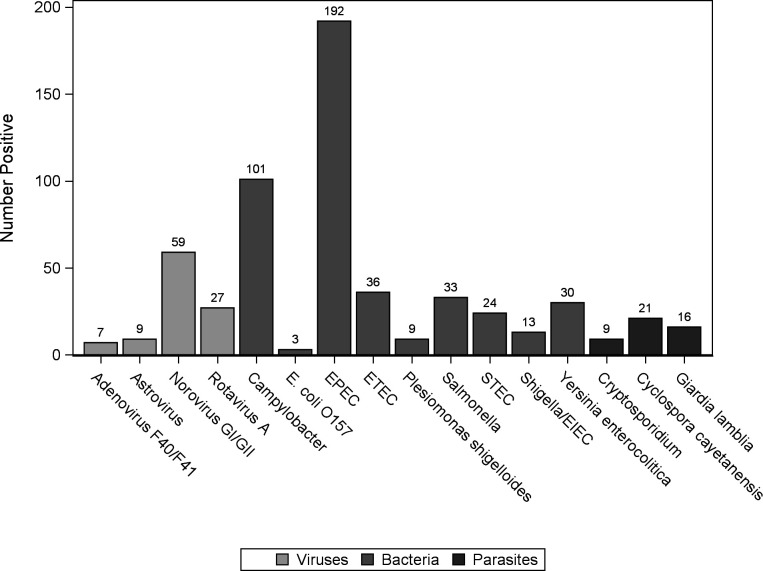
Positive fecal samples by enteric pathogens detected in stool samples examined using QIAstat-Dx Gastrointestinal Panel 2.

Among the prospective specimens tested, bacteria were the predominant microbe detected (74.87%, 441 cases), whereas viruses and parasites represented 17.32% (102 cases) and 7.81% (46 cases), respectively. The most prevalent microbe among all the prospective stool specimens examined is EPEC (192 cases), followed by *Campylobacter* (101 cases) and norovirus GI/GII (59 cases) (Fig. 1). Less than 40 cases were identified for each of the remaining microbes shown in Fig. 1. *E. coli* O157, only detected in three fecal specimens, was the pathogen with the lowest number of cases among all the targets investigated.

**Fig 2:**
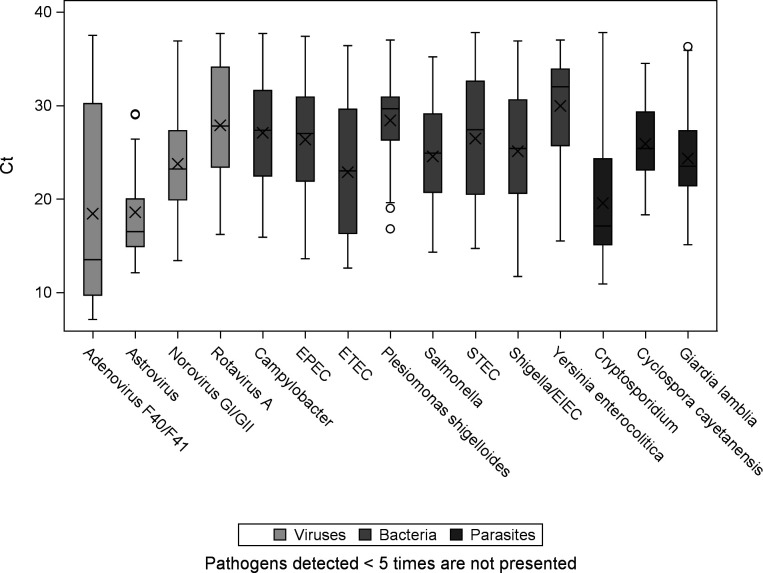
Ct values for the pathogens identified on the QIAstat-Dx Gastrointestinal Panel 2 system. Data for pathogens detected <5 times are not shown. x represents the mean; the lower quartile, median, and upper quartile are shown by the box; bars represent either the minimum and maximum values; and outliers are shown as circles. If outliers are present, the bars represent +/-1.5 x the interquartile range.

Most of the pathogens identified had C_T_ values in the range of 20–30. *Y. enterocolitica* displayed the highest median C_T_ value of 32, followed by *P. shigelloides* with a median C_T_ value of 29.65 (Fig. 2). Only three pathogens out of the 15 pathogens shown, namely, astrovirus, adenovirus F40/F41, and *Cryptosporidium*, demonstrated median C_T_ values below 20 each (Fig. 2).

### Detection of mixed infections in clinical prospective specimens

Within the population examined, co-infections were identified in 44 prospective specimens. This represents 11.5% of positive specimens (44/384). Most multiple detections contained two organisms (36/44; 91.8%), while 18.2% (8/44) contained three organisms. The analytes most commonly found in co-infections were EPEC (17), *Campylobacter* (16), norovirus GI/GII (12), ETEC (11), and *Plesiomonas shigelloides* (8) (Tables 4 and 5).

**TABLE 4:** Frequency of pathogen detections in co-infections as identified by QIAstat-Dx Gastrointestinal Panel 2 (in total 96 pathogen detections occurred among the 44 specimens showing mixed infections)

Analyte	N	%
Adenovirus F40/F41	2	2.1
*Astrovirus*	2	2.1
*Campylobacter*	16	16.7
*Cryptosporidium*	2	2.1
*Cyclospora cayetanensis*	2	2.1
Enteropathogenic *E. coli* (EPEC)	17	17.7
Enterotoxigenic *E. coli* (ETEC) *lt/st*	11	11.5
*Giardia lamblia*	3	3.1
Norovirus GI/GII	12	12.5
*Plesiomonas shigelloides*	8	8.3
Rotavirus A	5	5.2
*Salmonella*	4	4.2
Shiga-like toxin *E. coli* (STEC) *stx1/stx2*	5	5.2
*Shigella*/enteroinvasive *E. coli* (EIEC)	5	5.2
*Yersinia enterocolitica*	2	2.1
Total	96	100.0

**TABLE 5:** Most prevalent multiple detection combinations (≥2 instances) as determined by the QIAstat-Dx Gastrointestinal Panel 2

Multiple detection combination	Number of specimens
*Campylobacter* + norovirus GI/GII	2
Enterotoxigenic *E. coli* (ETEC) *lt/st* + norovirus GI/GII	3
*Campylobacter* + enteropathogenic E. coli (EPEC)	3
Enteropathogenic *E. coli* (EPEC) +*Salmonella*	3
*Campylobacter* + Rotavirus A	4
Enteropathogenic *E. coli* (EPEC) +Enterotoxigenic *E. coli* (ETEC) *lt/st*	4

Overall, for any given microbe identified in stool specimens tested, single infections dominated when compared to co-infections (Fig. S3A). Analysis of the patients’ age distribution for single versus co-infections demonstrated that co-infection cases predominate only in children between the ages of 0 and 6 (51.2% co-infections versus 48.8% single infections) (Fig. S3B). In other older age groups, single infections account for most cases (Fig. S3B).

### Performance evaluation of the QIAstat-Dx Gastrointestinal Panel 2

The individual performance of the prospective, retrospective, and prospectively collected and then archived positive clinical samples is shown in Tables 6 and 7 and Table 8, respectively. The combined results of the clinical performance evaluation from the prospective, prospectively collected and then archived positive clinical samples and retrospective studies are shown in Table 9. Of the 17 pathogens investigated, four pathogens showed a PPA value ≥95%: *Campylobacter* (97.73%; 95% CI: 93.50%–99.53%), *Cryptosporidium* (95.24%; 95% CI: 83.84%–99.42%), *C. cayetanensis* (95.83%; 95% CI: 78.88%–99.89%), and *G. lamblia* (100.00%; 95% CI: 94.31%–100.00%). Another 12 targets displayed PPA values between 90% and 95%: adenovirus F40/F41 (92.06%; 95% CI: 82.44%–97.37%), astrovirus (91.67%; 95% CI: 61.52%–99.79%), norovirus GI/GII (92.1%; 95% CI: 87.3%–95.1%), rotavirus A (91.89%; 95% CI: 78.09%–98.30%), *P. shigelloides* (90.91%; 95% CI: 78.33%–97.47%), *Salmonella* (94.12%; 95% CI: 85.62%–98.37%), *Y. enterocolitica* (94.44%; 95% CI: 84.61%–98.84%), EPEC (90.70%; 95% CI: 84.31%–95.10%), ETEC (92.6%; 95% CI: 83.9%–96.8%), STEC *stx1/stx2* (92.8%; 95% CI: 85.1%–96.6%), *E. coli* O157 (92.86%; 95% CI: 80.52%–98.50%), and EIEC (94.44%; 95% CI: 81.34%–99.32%). No *E. histolytica* was detected. Except for four targets—astrovirus, rotavirus A, *P. shigelloides*, and *C. cayetanensis*—the remaining 12 targets displayed lower 95% CI boundaries of >80%. All 17 targets displayed NPA values ≥ 95% each. Overall, the testing panel showed a PPA of 93.61% and an NPA of 99.77% for the 17 targets examined (Table 9). The panel showed a comparable performance when testing stool specimens collected either with Para-Pak C&S and FecalSwab (data not shown).

**TABLE 6:** Performance of the QIAstat-Dx Gastrointestinal Panel 2 as evaluated with prospective clinical specimens collected using FecalSwab or Para-Pak C&S medium before discordant analysis^*a*^

		Positive percent agreement			Negative percent agreement		
Analyte	Medium brand	TP/TP + FN	%	95%CI	TN/TN + FP	%	95%CI
Viruses							
Adenovirus F40/F41	FecalSwab	5/6	83.3	43.7–97.0	1,216/1,216	100.0	99.7–100.0
	Para-Pak C&S	1/2	50.0	9.5–90.6	703/704	99.9	99.2–100.0
Astrovirus	FecalSwab	3/3	100.0	43.9–100.0	1,219/1,219	100.0	99.7–100.0
	Para-Pak C&S	6/6	100.0	61.0–100.0	700/700	100.0	99.5–100.0
Norovirus GI/GII	FecalSwab	31/33	93.9	80.4–98.3	493/495	99.6	98.6–100.0
	Para-Pak C&S	14/18	77.8	54.8–91.0	399/399	100.0	99.1–100.0
Rotavirus A	FecalSwab	21/23	91.3	73.2–97.6	1,197/1,199	99.8	99.4–100.0
	Para-Pak C&S	3/3	100.0	43.9–100.0	702/703	99.9	99.2–100.0
Bacteria							
*Campylobacter*	FecalSwab	65/67	97.0	89.8–99.2	1,151/1,155	99.7	99.1–99.9
	Para-Pak C&S	30/31	96.8	83.8–99.4	675/677	99.7	98.9–99.9
*Plesiomonas shigelloides*	FecalSwab	0/0	N/A	N/A	1,220/1,222	99.8	99.4–100.0
	Para-Pak C&S	5/6	83.3	43.7–97.0	698/700	99.7	99.0–99.9
*Salmonella*	FecalSwab	14/16	87.5	64.0–96.5	1,206/1,206	100.0	99.7–100.0
	Para-Pak C&S	19/20	95.0	76.4–99.1	688/688	100.0	99.4–100.0
*Yersinia enterocolitica*	FecalSwab	15/16	93.8	71.7–99.0	1,199/1,206	99.4	98.8–99.7
	Para-Pak C&S	3/3	100.0	43.9–100.0	698/703	99.3	98.4–99.7
Diarrheagenic *E. coli*/*Shigella*							
Enteropathogenic *E. coli* (EPEC)	Para-Pak C&S	57/65	87.7	77.6–93.6	632/632	100.0	99.4–100.0
Enterotoxigenic *E. coli* (ETEC) *lt/st*	FecalSwab	9/10	90.0	59.6–99.2	427/430	99.3	98.0–99.8
	Para-Pak C&S	9/10	90.0	59.6–99.2	390/395	98.7	97.1–99.5
Shiga-like toxin *E. coli* (STEC) *stx1*/*stx2*	Para-Pak C&S	5/6	83.3	43.7–97.0	397/400	99.3	97.8–99.7
*E. coli* O157	Para-Pak C&S	0/0	N/A	N/A	5/5	100.0	56.6–100.0
*Shigella*/enteroinvasive *E. coli* (EIEC)	FecalSwab	10/10	100.0	72.3–100.0	1,212/1,212	100.0	99.7–100.0
	Para-Pak C&S	2/2	100.0	34.2–100.0	703/704	99.9	99.2–100.0
Parasites							
*Cryptosporidium*	FecalSwab	2/4	50.0	15.0–85.0	1,218/1,218	100.0	99.7–100.0
	Para-Pak C&S	6/6	100.0	61.0–100.0	699/700	99.9	99.2–100.0
*Cyclospora cayetanensis*	FecalSwab	3/3	100.00	43.9–100.0	1,219/1,219	100.0	99.7–100.0
	Para-Pak C&S	18/19	94.7	75.4–99.1	687/687	100.0	99.4–100.0
*Entamoeba histolytica*	FecalSwab	0/0	N/A	N/A	1,222/1,222	100.0	99.7–100.0
	Para-Pak C&S	0/0	N/A	N/A	706/706	100.0	99.5–100.0
*Giardia lamblia*	FecalSwab	6/8	75.0	40.9–92.9	434/441	98.4	96.8–99.2
	Para-Pak C&S	1/1	100.0	20.7–100.0	406/406	100.0	99.1–100.0

^*a*^ TP, true positive; TN, true negative; FP, false positive; FN, false negative; CI, confidence interval, N/A, not available.

**TABLE 7:** Performance of the QIAstat-Dx Gastrointestinal Panel 2 as evaluated with clinical retrospective specimens collected using FecalSwab or Para-Pak C&S medium before discordant analysis^*a*^

		Positive percent agreement			Negative percent agreement		
Analyte	Medium brand	TP/TP + FN	%	95%CI	TN/TN + FP	%	95%CI
Viruses							
Adenovirus F40/F41	FecalSwab	23/26	88.5	71.0–96.0	203/203	100.0	98.1–100.0
	Para-Pak C&S	29/29	100.0	88.3–100.0	39/39	100.0	91.0–100.0
Astrovirus	FecalSwab	2/3	66.7	20.8–93.9	191/191	100.0	98.0–100.0
	Para-Pak C&S	0/0	N/A	N/A	14/14	100.0	78.5–100.0
Norovirus GI/GII	FecalSwab	28/32	87.5	71.9–95.0	74/75	98.7	92.8–99.8
	Para-Pak C&S	27/29	93.1	78.0–98.1	86/86	100.0	95.7–100.0
Rotavirus A	FecalSwab	8/9	88.9	56.5–98.0	185/185	100.0	98.0–100.0
	Para-Pak C&S	2/2	100.0	34.2–100.0	12/12	100.0	75.8–100.0
Bacteria							
*Campylobacter*	FecalSwab	31/31	100.0	89.0–100.0	161/163	98.8	95.6–99.7
	Para-Pak C&S	3/3	100.0	43.9–100.0	11/11	100.0	74.1–100.0
*Plesiomonas shigelloides*	FecalSwab	2/2	100.0	34.2–100.0	192/192	100.0	98.0–100.0
	Para-Pak C&S	33/36	91.7	78.2–97.1	117/117	100.0	96.8–100.0
*Salmonella*	FecalSwab	30/31	96.8	83.8–99.4	161/163	98.8	95.6–99.7
	Para-Pak C&S	1/1	100.0	20.7–100.0	13/13	100.0	77.2–100.0
*Yersinia enterocolitica*	FecalSwab	32/34	94.1	80.9–98.4	160/160	100.0	97.7–100.0
	Para-Pak C&S	1/1	100.0	20.7–100.0	14/14	100.0	78.5–100.0
Diarrheagenic *E. coli*/*Shigella*							
Enteropathogenic *E. coli* (EPEC)	Para-Pak C&S	60/65	92.3	83.2–96.7	42/42	100.0	91.6–100.0
Enterotoxigenic *E. coli* (ETEC) *lt/st*	FecalSwab	22/24	91.7	74.2–97.7	85/86	98.8	93.7–99.8
	Para-Pak C&S	23/24	95.8	79.8–99.3	61/61	100.0	94.1–100.0
Shiga-like toxin *E. coli* (STEC) *stx1/stx2*	Para-Pak C&S	60/64	93.8	85.0–97.5	44/44	100.0	92.0–100.0
*E. coli* O157	Para-Pak C&S	39/42	92.9	80.1–99.4	16/16	100.0	80.6–100.0
*Shigella*/Enteroinvasive *E. coli* (EIEC)	FecalSwab	22/24	91.7	74.2–97.7	170/170	100.0	97.8–100.0
	Para-Pak C&S	0/0	N/A	N/A	14/14	100.0	78.5–100.0
Parasites							
*Cryptosporidium*	FecalSwab	6/6	100.0	61–100.0	186/188	98.9	96.2–99.7
	Para-Pak C&S	26/26	100.0	87.1–100.0	117/117	100.0	96.8–100.0
*Cyclospora cayetanensis*	FecalSwab	1/1	100.0	20.7–100.0	193/193	100.0	98.1–100.0
	Para-Pak C&S	1/1	100.0	20.7–100.0	13/13	100.0	77.2–100.0
*Entamoeba histolytica*	FecalSwab	0/0	N/A	N/A	194/194	100.0	98.1–100.0
	Para-Pak C&S	0/0	N/A	N/A	14/14	100.0	76.5–100.0
*Giardia lamblia*	FecalSwab	29/31	93.6	79.3–98.2	46/48	95.8	86.0–98.9
	Para-Pak C&S	27/28	96.4	82.3–99.4	92/92	100.0	96.0–100.0

^*a*^ TP, true positive; TN, true negative; FP, false positive; FN, false negative; CI, confidence interval, N/A, not available.

**TABLE 8:** Performance of the QIAstat-Dx Gastrointestinal Panel 2 as evaluated with prospectively collected and then archived positive clinical samples collected using FecalSwab or Para-Pak C&S medium before discordant analysis^*a*^

		Positive percent agreement			Negative percent agreement		
Analyte	Medium brand	TP/TP + FN	%	95%CI	TN/TN + FP	%	95%CI
Norovirus GI/GII	FecalSwab	48/49	98.0	89.3–99.6	2/4	50.0	15.0–85.0
	Para-Pak C&S	26/28	92.9	77.4–98.0	37/38	97.4	86.5–99.5
Shiga-like toxin *E. coli* (STEC) *stx1/stx2*	Para-Pak C&S	12/13	92.3	66.7–98.6	51/52	98.1	89.9–99.7

^*a*^ TP, true positive; TN, true negative; FP, false positive; FN, false negative; CI, confidence interval.

**TABLE 9:** Overall performance of the QIAstat-Dx Gastrointestinal Panel 2 as evaluated with clinical specimens (prospective, prospectively collected and then archived positive clinical samples and retrospective) collected using FecalSwab or Para-Pak C&S medium before discordant analysis^*a*^

	Positive percent agreement			Negative percent agreement		
Analyte	TP/TP + FN	%	95% CI (%)	TN/TN + FP	%	95% CI (%)
Viruses						
Adenovirus F40/F41	58/63	92.06	82.44–97.37	2159/2160	99.95	99.74–100.00
Astrovirus	11/12	91.67	61.52–99.79	2,124/2,124	100.00	99.83–100.00
Norovirus GI/GII	174/189	92.1	87.3–95.1	1,091/1,097	99.45	98.81–99.80
Rotavirus A	34/37	91.89	78.09–98.30	2,096/2,099	99.86	99.58–99.97
Bacteria						
*Campylobacter*	129/132	97.73	93.50–99.53	1,998/2,006	99.60	99.22–99.83
*Plesiomonas shigelloides*	40/44	90.91	78.33–97.47	2,227/2,231	99.82	99.54–99.95
*Salmonella*	64/68	94.12	85.62–98.37	2,068/2,070	99.90	99.65–99.99
*Yersinia enterocolitica*	51/54	94.44	84.61–98.84	2,071/2,083	99.42	99.00–99.70
Diarrheagenic *Escherichia coli*/Shigella						
Enteropathogenic *Escherichia coli* (EPEC)	117/130	90.00	84.51–94.57	674/674	100.00	99.45–100.00
Enterotoxigenic *Escherichia coli* (ETEC) *lt/st*	63/68	92.6	83.9–96.8	963/972	99.07	98.25–99.58
Shiga-like toxin *Escherichia coli* (STEC) *stx1/stx2*	77/83	92.8	85.1–96.6	492/496	99.19	97.95–99.78
*Escherichia coli* O157	39/41	95.12	83.47–99.40	21/21	100.00	83.89–100.00
Shigella/enteroinvasive *Escherichia coli* (EIEC)	34/36	94.44	81.34–99.32	2,099/2,100	99.95	99.73–100.00
Parasites						
*Cryptosporidium*	40/42	95.24	83.84–99.42	2,220/2,223	99.87	99.61–99.97
*Cyclospora cayetanensis*	23/24	95.83	78.88–99.89	2,112/2,112	100.00	99.83–100.00
*Entamoeba histolytica*	0/0	N/A	N/A	2,136/2,136	100.00	99.83–100.00
*Giardia lamblia*	63/63	100.00	94.31–100.00	983/993	98.99	98.16–99.52
Overall	981/1,048	93.61%	91.95%–95.01%	27,534/27,597	99.77%	99.71%–99.82%

^*a*^ TP, true positive; TN, true negative; FP, false positive; FN, false negative; CI, confidence interval, N/A, not available.

### Discrepancy analysis

A total of 43 false negatives (FN) were identified in this investigation. Measurement with an alternative comparator assay revealed that 27 of these FNs were also negative by the alternate method, while 12 were positive (confirming the FN QIAstat result) and 4 cases unresolved (Table S2). Of the 34 false positives (FPs) detected, six FPs were found to be positive by the alternate comparator, whereas 28 FPs were not found positive by the alternative method (Table S2). *Campylobacter* had the largest number of FPs confirmed to be positive by an alternate method (5 out of 8 FP cases (Table S2).

## DISCUSSION

We assessed the performance of the QIAstat-Dx Gastrointestinal Panel 2 through testing stool specimens in modified Cary-Blair transport medium in a multicenter clinical laboratory study. Two stool specimen collection methods were assessed: Para-Pak C&S and FecalSwab. This multiplex PCR syndromic panel exhibited high PPA and NPA in its ability to identify 17 different pathogens involved in gastrointestinal infection disease. Overall, the panel demonstrated PPA values > 90% and NPA values > 98% for each pathogen detected and showed comparable performance when testing stool specimens collected either with Para-Pak C&S and FecalSwab.

The three most common pathogens identified in this investigation among prospective specimens were EPEC (192 cases), *Campylobacter* (101 cases), and norovirus GI/GII (59 cases). These three pathogens are among the six most common pathogens causing gastrointestinal infections and were detected by other research groups in the United States (23, 24) and in Europe (25) who utilized multiplex PCR panel-based testing. EPEC infections, which occur at a higher rate in children than in adults, account for 5%–10% of pediatric diarrhea cases in the developing world (26). EPEC is also highly prevalent in high-income countries, with EPEC infections accounting for 29.5% of the total pathogens detected in an investigation carried out in the United States (23). EPEC is commonly present in co-infections with other gastrointestinal pathogens, and sometimes it presents as a bystander during infection (27, 28). The large number of *Campylobacter* cases detected in this study is not surprising given a surge in *Campylobacter* infections in recent years in North America and Europe (29, 30). Both *C. jejuni* and *C. coli* are established causes of diarrhea in humans, whereas *C. upsaliensis* is considered an emerging pathogen in gastroenteritis (29). Norovirus, the third-most common pathogen identified in this investigation, is associated with approximately 18% of diarrheal cases globally, with a higher incidence in children aged <5 years and in adults aged ≥65 years (31). Norovirus infections are associated with approximately 1 million healthcare visits per year, and following the implementation of rotavirus vaccination, norovirus has emerged as the leading etiologic agent for U.S. children with medically attended acute gastroenteritis (32).

Unlike conventional methods such as culture, microscopy, and antigen testing, the multiplex PCR approach is more sensitive. It has the potential to detect more than one target in a single test. However, as pointed out by some guidelines, culture may still be necessary for antimicrobial susceptibility and public health surveillance (33, 34). In this study, co-infections were detected in 44 prospective stool specimens or 11.4% of total prospective stool specimens with at least two pathogens detected. These findings are similar to those of a study that reported mixed infections in 21.1% and 13.0% of positive prospective samples, as detected by FilmArray GI panel and Luminex xTAG Gastrointestinal Pathogen Panel, respectively, compared to 8.3% by conventional techniques (35). In another study conducted at a U.S. Midwestern academic hospital, 19.3% of the total positive fecal samples exhibited mixed infections, with EPEC, norovirus, and ETEC among the top five most common pathogens identified in co-infections (24).

The ability of multiplex PCR panels such as QIAstat-Dx Gastrointestinal Panel 2 to detect co-infections that might otherwise be missed may help clinicians to make more informed treatment decisions and improve patient outcomes as it can predict a more severe clinical outcome (36); however, these results must be interpreted with caution. It is likely that in a patient with mixed infections, one pathogen may be the major pathogen that determines the clinical outcome. Given that the QIAstat-Dx Gastrointestinal Panel 2 measures amplification curves and the corresponding C_T_ values for each pathogen detected, the C_T_ values could be further studied in the future to determine if these values correlate directly with current pathogen load. Some studies show a relation between the pathogens’ load in the specimens, which can be reflected by the C_T_ values, and the pathogenic role of the microorganism (37, 38).

However, it is well established that enteropathogens are present in symptomatic as well as asymptomatic persons. An investigation carried out on children attending day care centers in the Netherlands revealed that enteropathogens of bacterial, parasitic, and viral origin were identified in 78.0% of 5,197 stool samples—95.4% of which were collected from children who did not display any gastroenteritis symptoms—and found that only norovirus and rotavirus were significantly associated with gastroenteritis among the children attending day care centers (39). Furthermore, other studies have reported the excretion of enteropathogens for extended periods of time. Studies have reported that rotavirus is excreted for up to 57 days after the onset of diarrhea in children (40), and norovirus shedding lasts up to 56 days post-infection in healthy adults (41).

This study has several limitations. The LoD for each pathogen may vary considerably between the various multiplex PCR panels. This difference could be due to different primers and probes used by the various multiplex PCR panels to amplify a particular target. Different primers display different binding kinetics to the target nucleic acid, resulting in different amplification efficiencies. Due to the small number of positive specimens collected for certain analytes (e.g., *E. coli* 0157) during the prospective clinical study, performance characteristics were established additionally with retrospective clinical specimens. For targets that were not identified in sufficient numbers (at least 50 positive results) during testing with prospective and retrospective clinical specimens, we relied on testing of contrived samples to assess the performance of the QIAstat-Dx Gastrointestinal Panel 2 (refer to Supplemental Methods for details, Table S3 and S4). Positive percent agreement (PPA) for *Entamoeba histolytica* was established only with contrived clinical specimens, while negative percent agreement was established on clinical specimens. For the four targets where a composite comparator was used (norovirus, ETEC, STEC, and *Giardia lamblia*), the discrepancies were not further investigated. Given that the stool specimens studied were obtained from healthcare centers in the United States and Europe, the prevalence of the pathogens detected may not reflect the prevalence of other geographic regions. Furthermore, epidemiological data (coming from the prospective study arm) are limited to the inclusion criteria of the study design.

In conclusion, our multicenter assessment of the QIAstat-Dx Gastrointestinal Panel 2 revealed that this testing system displayed high PPA and NPA in detecting 17 targets of bacterial, viral, and parasitic origin. The system allows reporting of Ct values, which might provide additional research insights on the infection status and be of particular interest in the context of co-infections. QIAstat-Dx Gastrointestinal Panel 2 provides a new alternative for multiplex gastrointestinal testing that, with its accurate and robust assay, allows for the rapid and comprehensive testing of gastrointestinal infections.

## Data Availability

The data sets generated during the current study are available from the corresponding author on reasonable request.

## SUPPLEMENTAL MATERIAL

Supplemental materials jcm.01983-24-s0001.docx Supplemental methods, Fig. S1 to S3B, Tables S1 to S4, and supplemental references.

Figure S1 jcm.01983-24-s0002.tiff Median and quartiles of age of patients whose stool samples tested positive for various pathogens on the QIAstat-Dx Gastrointestinal Panel 2.

Figure S2 jcm.01983-24-s0003.tiff Source of the pathogens according to patients' status within the healthcare system. Pathogens detected <5 times are not presented.

Figure S3A jcm.01983-24-s0004.tiff Frequency of single and co-infections for the various pathogens as detected following testing on the QIAstat-Dx Gastrointestinal Panel 2.

Figure S3B jcm.01983-24-s0005.tiff Percentages of single and co-infections within the various age groups of patients.

ASM does not own the copyrights to Supplemental Material that may be linked to, or accessed through, an article. The authors have granted ASM a non-exclusive, world-wide license to publish the Supplemental Material files. Please contact the corresponding author directly for reuse.
