# Supplementary material for: Multicenter evaluation of the QIAstat-Dx Gastrointestinal Panel 2, a multiplex PCR platform for the diagnosis of acute gastroenteritis
Source: J Clin Microbiol. 2025 Jul 11;63(8):e01983-24. doi: 10.1128/jcm.01983-24 (PMC12345276; doi:10.1128/jcm.01983-24)
Supplement: Supplemental materials — Supplemental methods, Fig. S1 to S3B, Tables S1 to S4, and supplemental references. [file jcm.01983-24-s0001.docx]

# Supplemental Methods

## External controls testing on QIAstat-Dx Gastrointestinal Panel 2

External control testing was conducted as a mean of ensuring the integrity of the results obtained with the clinical specimens and supporting assay performance. Toward this end, these external controls were generated as 4 different mixes of pathogens to serve as quality control samples:

Mix 1: Astrovirus, *C. cayetanensis*, *C. jejuni* and *Shigella* (*Shigella sonnei*).

Mix 2: Norovirus GI, EPEC, *Y. enterocolitica*, *P. shigelloides*, Adenovirus type 41, and rotavirus.

Mix 3: *G. lamblia*, *E. histolytica*, ETEC, and *Salmonella* (*S. enterica Typhimurium*)*.*

Mix 4: Norovirus GII, *Cryptosporidium* (*C. parvum*), and STEC O157.

All pathogens used to prepare the external control samples were purchased from ZeptoMetrix (NATtrol Gastrointestinal Verification Panel) and these pathogens have been rendered non-infectious through chemical modifications. A negative clinical control (Mix 5) was also prepared using a non-infected person’s stool specimen resuspended in modified Cary-Blair medium. The positive and negative mixes were prepared in bulk and subsequently aliquoted into tubes containing 220 μl (for single use) prior to storage in freezers between −15°C and −30°C. Each sample was only subjected to a single freeze and thaw cycle.

## Composite comparator method justification

Note that a composite comparator method was used in some cases due to data available suggesting that the FilmArray™ GI panel needed further support as comparator to accurately establish the performance characteristics of the investigational device.

- Data from the clinical study supporting clearance of the FilmArray™ GI panel reported that 10/18 (55.55%) false positive for Norovirus GII/GII against the comparator CDC CaliciNet assays were not detected using bi-directional analyses during discrepancy investigation (1).
- The manufacturer (BioMérieux) issued a field safety corrective action (FSCA) on January 2024 to inform customers of a product recall (correction) involving the FilmArray™ GI panel due to the identified potential signal of increased false positive Norovirus results (FSCA 5812).
- A recent publication from Caza et al., 2024 has reported suspected false positive norovirus results (27.4% of positives) after introduction of the FilmArray™ GI panel into six laboratories (British Columbia, Canada) (2). In line, Matic et al., 2024 reported a higher-than-expected number of stool samples with norovirus when using FilmArray™ GI panel in their clinical laboratory, resulting in investigations reporting 36% of these positives being negative with another molecular test (Xpert® Norovirus, Cepheid) (3).
- BioMérieux updated the Instructions for Use (IFU) of the FilmArray™ GI panel in November 2024. The update includes additional clinical data from 2023 related to a post-marketing performance follow-up study, which includes changes in specificity for Norovirus results. The updated performance data confirms FilmArray™ GI panel still presents a false positive rate of 42.6% for Norovirus GI/GII. Cross-reactivity has been observed with several off-panel organisms, contributing to these false positives.
- Published data from the multicenter clinical evaluation of the FilmArray™ GI panel (1) used to support its clearance (#K140407) showed ETEC false positives (9.7% of all ETEC detections) caused by cross-reactivity between the ETEC assay primers and commensal organisms (*Citrobacter koseri* and *Hafnia alvei*) that contain variants of the *fliP* gene. This potential for false-positive ETEC results for the FilmArray™ GI panel is noted in the Limitations of the Procedure section of the FilmArray™ GI panel package insert which details that certain strains of *Citrobacter koseri, Citrobacter sedlakii, Hafnia alvei*, and *Cedeceae davisiae* containing variants of a flagellar assembly protein have the potential to cross-react with FilmArray™ GI panel ETEC 2 assay.
- According to the *in silico* analyses documented in the Analytical Reactivity Section from the FilmArray™ GI panel 510(k) Decision Summary (#K140407), STEC stx2 subtype f is predicted to be detected with reduced sensitivity or not detected by the FilmArray™ GI panel STEC assays.
- In line, Cointe et al., 2020 reported a false-negative result of STEC stx2f when using FilmArray™ GI panel in their clinical laboratory (4).
- Published data from the multicenter clinical evaluation of the FilmArray™ GI panel (1) used to support its clearance (#K140407) showed *Giardia lamblia* false positives (7.4% of all *Giardia lamblia* detections) caused by cross-reactivity of the *Giardia lamblia* assay primers to certain commensal organisms (*Bifidobacterium longum* and *Ruminococcus callidus*). This potential for false-positive *Giardia lamblia* is noted in the Limitations of the Procedure section of the FilmArray™ GI panel package insert which details that *Bifidobacterium spp*. and *Ruminococcus spp.* have the potential to cross-react with the FilmArray™ GI panel *Giardia lamblia* assay.

Provided with the overall body of evidence stated above, it was considered that for these targets the performance using a composite approach provided a more accurate performance estimate of the device and prove its safety and effectiveness to diagnose the corresponding analytes in stool samples.

## Multiplex PCR assay using FilmArray™ GI panel

Testing with this panel was performed according to the manufacturer’s instructions ([www.biofiredx.com/wp-content/uploads/2018/05/FLM1-MKT-0071-FilmArray-GI-Procedure-CLSI-Format.pdf](http://www.biofiredx.com/wp-content/uploads/2018/05/FLM1-MKT-0071-FilmArray-GI-Procedure-CLSI-Format.pdf)). Briefly, a BioFire GI Panel pouch was placed into a pouch loading station. This pouch was then hydrated with hydration solution. To prepare the sample, stool sample suspended modified Cary-Blair medium was mixed thoroughly. A total of 0.2 mL of this suspension was then transferred to a Sample Injection Vial and mixed well. The sample was subsequently introduced into the BioFire GI Panel pouch by inserting the Sample Injection Vial into the pouch sample port. The loaded pouch is then inserted into either a BioFire FilmArray 2.0 or a BioFire FilmArray Torch instrument before starting the test run.

## Testing with BD MAX system

A total of 3 different panels from the BD MAX Enteric Panel testing system (BD MAX Enteric Viral Panel, BD MAX Enteric Bacteria Panel, and BD MAX Extended Enteric Bacteria Panel) were utilized as a comparator method to identify adenovirus F40/F41, norovirus GI/GII, ETEC, and STEC *stx1/stx2*. The assays were performed according to the manufacturer’s instructions (BD MAX Enteric Viral Panel, BD MAX Enteric Bacteria Panel and BD MAX Extended Enteric Bacteria Panel). In short, a large disposable inoculation loop was inserted into a stool sample suspended in modified Cary-Blair. The loop was then transferred into a Sample Buffer Tube and swirled to mix its contents. The Sample Buffer Tube was subsequently loaded onto the BD MAX instrument together with the panel’s Unified Reagent Strip, which has tubes for nucleic acid extraction and PCR reagent mix, and a PCR cartridge. The BD MAX instrument has the capacity to process and test up to 24 samples per test run. Following the loading of the samples and PCR cartridge, the test was initiated. Cell lysis, nucleic acid isolation, target amplification, and detection with real-time PCR steps were executed within the BD MAX instrument.

## Testing with Luminex xTAG Gastrointestinal Pathogen Panel

This multiplex PCR panel-based testing system was used as part of a composite of 3 comparator methods to detect adenovirus F40/F41, norovirus GI/GII, ETEC, and STEC *stx1/stx2*. Testing was carried out according to the manufacturer’s recommendations.

Prior to purifying nucleic acids from the clinical stool specimens resuspended in modified Cary-Blair medium, stool specimens were subjected to treatment with lysis buffer and bead beating to maximize extraction efficiency. For each stool sample placed in a tube containing lysis buffer and beads, 10 μl of xTAG MS2 bacteriophage was added to the tube as a positive control. Following lysis treatment, the tubes were centrifuged and 200 μl of supernatant was collected from each tube for nucleic acid isolation. Nucleic acids were isolated using NucliSENS easyMAG (bioMerieux) or QIAamp minElute Virus Spin Kit (QIAGEN). For each clinical specimen, a total of 10 μl of purified nucleic acid sample was added to a 15 μl master mix containing xTAG Rnase-free water, xTAG OneStep buffer, xTAG Gastrointestinal Pathogen Panel primer mix, xTAG BSA, and xTAG OneStep Enzyme Mix. Tubes containing the resulting mixture were placed in a thermal cycler and underwent the following cycling conditions: 53°C (20 minutes) for 1 cycle; 95°C (15 minutes) for 1 cycle; 95°C (30 seconds), 58°C (30 seconds), and 72°C (30 seconds) for 38 cycles; 72°C (2 minutes) for 1 cycle. The tubes were on hold at 4°C until further analysis was performed. A total of 5 μl of the RT-PCR product from each sample was added to well of a 96-well microtiter plate containing 20 μl of the xTAG Gastrointestinal Pathogen Panel Bead Mix. Then, 75 μl of reporter solution (xTAG 0.22 SAPE in xTAG Reporter Buffer) was added to each well and mixed. The plate was placed in a thermal cycler and subjected to the following temperatures: 60°C for 3 minutes, 45°C for 45 minutes, and finally on hold at 45°C. Detection of bead hybridization and data analysis was carried out using a Luminex 100/200 or MAGIPIX instrument.

## PCR followed by BDS (bi-directional sequencing)

All PCR-BDS assays used as comparator or discrepancy in the study have been validated as follows.

*Validation of the assay for identifying G. duodenalis*

The two PCR assays were developed by Qiagen and both target different regions of the following gene: Beta-giardin gene (see for reference NCBI Gene ID: 5698276). Each PCR assay was validated by evaluating the assay Limit of Detection (LoD) using whole organism (culture isolate ATCC 30957) spiked in stool matrix (negative stools preserved in Cary-Blair) and each replicate test included all processing steps from sample extraction, following PCR and bi-directional sequencing. The PCR assays limit of detection (LoD) was tested in serial dilutions (20 replicates each) and was shown to be at least as sensitive as the QIAstat-Dx LoD. In addition, precision and robustness for each of the assays were confirmed by execution of a repeatability (intra-variability) and reproducibility (inter-variability) study where negative and positive samples (1 x LoD previously determined) were tested using 15 replicates. Finally, *in silico* sequence analyses were conducted to evaluate the reactivity and specificity of the two PCR assays. The primers sequences analytical reactivity was confirmed by analyzing 252 and 449 sequences retrieved from NCBI database for each of the two assays, respectively. The primers sequences analytical specificity was confirmed by BLAST searches against the nt NCBI database excluding the taxid: 5741 (Giardia lamblia).

*Validation of the assay for identifying EPEC*

EPEC assay was developed by Genewiz and was validated as follows: The limit of detection was tested in serial dilutions (3 replicates each) and was shown to be at least as sensitive as the QIAstat-Dx. The robustness of the assay was evaluated by two additional operators by testing triplicates of the samples at the assay LoD concentration on different days. Sequencing traces from the LoD and robustness test were aligned against the reference sequence and used to generate consensus sequences. Qiagen performed additional *in silico* sequence analyses to evaluate the reactivity and specificity. The primers sequences analytical reactivity was confirmed by analyzing 1984 sequences retrieved from NCBI database. The primers sequences analytical specificity was confirmed by BLAST searches against the nt NCBI database excluding the taxid: 562 (*Escherichia coli*). The target gene for EPEC is the same in the BioFire, QIAstat-Dx and PCR-BDS assay (intimin gene (*eae*)).

*Validation of the assay for identifying Cryptosporidium*

*Cryptosporidium* assay was developed by Genewiz and was validated as follows: The limit of detection was tested in 21 replicates and was shown to be at least as sensitive as the QIAstat-Dx. The robustness of the assay was evaluated by two operators over at least 3 days by testing 21 replicates of the samples at the assay LoD concentration. Negative stools samples were also tested that supported the assay analytical specificity. Qiagen performed additional *in silico* sequence analyses to evaluate the reactivity and specificity. The primers sequences analytical reactivity was confirmed by analyzing 88 sequences retrieved from NCBI database. The primers sequences analytical specificity was confirmed by BLAST searches against the nt NCBI database excluding the taxid: 5807 / 237895 / 83540 / 93969 / 195482 (*Cryptosporidium* species).

## Contrived samples preparation

Contrived samples were also prepared for 15 microbial targets to ensure that positive samples were included within the study in sufficient numbers. These 15 pathogenic targets are adenovirus, astrovirus, *C. jejuni, Cryptosporidium, C. cayetanensis, E. coli* O157, EIEC, ETEC, *E. histolytica, G. lamblia, P. shigelloides,* rotavirus A, STEC stx1/stx2, *Salmonella spp.* and *Y. enterocolitica*.

The pathogens strains used to prepare contrived samples were purchased from commercial sample collections as culture isolates (Table S3) and were prepared at 2× Limit of Detection (LoD) (50% of contrived samples), 5× LoD, and 10× LoD by serial dilution of the stock culture isolates using artificial stool diluent (ZeptoMetrix). LoD was established during the analytical verification of the assay as detailed in the Analytical Performance section from the manufacturer’s instructions (https://www.qiagen.com/us/products/diagnostics-and-clinical-research/infectious-disease/qiastat-dx-syndromic-testing/qiastat-dx-na?catno=691421). These diluted samples were spiked into individual negative clinical stool samples in Cary Blair using FecalSwab or Para-Pak C&S. The contrived samples were blinded and randomized with clinical specimens or mixed with an equal number of known negative specimens. The contrived samples were distributed equally to all 13 clinical testing sites in EU and USA.

The negative stool specimens in Cary Blair (FecalSwab or Para-Pak C&S) used in the preparation of the contrived samples were obtained from EU or USA clinical settings. To confirm that these samples were negative for any of the targets tested, they were assayed on the FilmArray™ GI panel and the QIAstat-Dx system using the QIAstat-Dx Gastrointestinal Panel 2.

# Supplemental Figures

## Figure S1. Median and quartiles of age of patients whose stool samples tested positive for various pathogens on the QIAstat-Dx Gastrointestinal Panel 2. Pathogens detected <5 times are not presented. x represents the mean, the lower quartile, median and upper quartile are shown by the box, bars represent either the minimum and maximum values and outliers are shown as circles. If outliers are present the bars represent +/-1.5x the interquartile range.

## Figure S2. Source of the pathogens according to patients’ status within the healthcare system. Pathogens detected <5 times are not presented.

## Figure S3A. Frequency of single and co-infections for the various pathogens as detected following testing on the QIAstat-Dx Gastrointestinal Panel 2. Data for pathogens detected <5 times are not shown.

## Figure S3B. Percentages of single and co-infections within the various age groups of patients. Data for pathogens identified <5 times are not presented.

# Supplementary Tables

## Table S1. QIAstat-Dx® Gastrointestinal Panel 2 clinical studies comparator methods and discordant samples testing.

| **QIAstat-Dx^®^ GI2 Panel Test Result** | **Comparator Methods** | **Discordant testing method** |
| --- | --- | --- |
| Adenovirus F40/F41 | BIOFIRE® FILMARRAY® GI Panel | BD MAX™ Enteric Viral Panel |
| Astrovirus |  |  |
| Rotavirus A |  |  |
| *Campylobacter (C. jejuni, C. coli* and *C. upsaliensis)* |  | BD MAX™ Enteric Bacterial Panel |
| *Salmonella* |  |  |
| *Shigella/*Enteroinvasive *E. coli* (EIEC) |  |  |
| *Plesiomonas shigelloides* |  | BD MAX™ Extended Enteric Bacterial Panel |
| *Yersinia enterocolitica* |  |  |
| *E. coli* O157 |  | Luminex xTAG GPP |
| Enteropathogenic *E. coli* (EPEC) |  | PCR-BDS |
| *Cryptosporidium* |  |  |
| *Cyclospora cayetanensis* |  | NA |
| *Entamoeba histolytica* |  |  |
| Norovirus GI/GII | *Composite of three FDA-cleared/CE-marked test methods (BIOFIRE^®^ FILMARRAY^®^ GI Panel, Luminex xTAG^®^ GPP, BD MAX^TM^ Enteric Panels)* |  |
| Enterotoxigenic *E. coli* (ETEC) lt/st |  |  |
| Shiga-like toxin- *E. coli* (STEC) stx1/stx2 |  |  |
| *Giardia lamblia* | *Composite of two FDA-cleared/CE-marked test methods (BIOFIRE^®^ FILMARRAY^®^ GI Panel, Luminex xTAG^®^ GPP) and two validated PCR tests followed by bi-directional sequencing (PCR/BDS)* |  |

## Table S2. Discrepancy study results for prospective and retrospective clinical specimens assayed on QIAstat-Dx Gastrointestinal Panel 2.

| **Target pathogen** | **Total number of discrepant specimens** | **No. discrepant specimens tested** | **Outcome of discrepancy analysis (number of cases)** | | |
| --- | --- | --- | --- | --- | --- |
|  |  |  | **Not Analyzed** | **Result of QIAstat-Dx Gastrointestinal Panel 2** | |
|  |  |  |  | **Correct** | **Incorrect** |
| **False negatives** | | | | | |
| Adenovirus F40/41 | 5 | 5 | 0 | 4 | 1 |
| Astrovirus | 1 | 1 | 0 | 0 | 1 |
| Rotavirus A | 3 | 3 | 0 | 1 | 2 |
| *Campylobacter* | 3 | 3 | 0 | 3 | 0 |
| *Plesiomonas shigelloides* | 4 | 4 | 0 | 3 | 1 |
| *Salmonella* | 4 | 4 | 0 | 4 | 0 |
| *Yersinia enterocolitica* | 3 | 3 | 0 | 3 | 0 |
| Enteropathogenic *Escherichia coli* (EPEC) | 12 | 10 | 2 | 3 | 7 |
| *Escherichia coli* O157 | 3 | 2 | 1 | 2 | 0 |
| *Shigella*/Enteroinvasive *Escherichia coli* (EIEC) | 2 | 2 | 0 | 1 | 1 |
| *Cryptosporidium* | 2 | 2 | 0 | 2 | 0 |
| *Cyclospora cayetanensis* | 1 | 0 | 1 | 0 | 0 |
| Total | 43 | 39 | 4 | 27 | 12 |
| **False positives** | | | | | |
| Adenovirus F40/41 | 1 | 1 | 0 | 0 | 1 |
| Rotavirus A | 3 | 3 | 0 | 0 | 3 |
| *Campylobacter* | 8 | 8 | 0 | 5 | 3 |
| *Plesiomonas shigelloides* | 4 | 4 | 0 | 0 | 4 |
| *Salmonella* | 2 | 2 | 0 | 0 | 2 |
| *Yersinia enterocolitica* | 12 | 12 | 0 | 0 | 12 |
| *Shigella*/Enteroinvasive *Escherichia coli* (EIEC) | 1 | 1 | 0 | 1 | 0 |
| *Cryptosporidium* | 3 | 3 | 0 | 0 | 3 |
| Total | 34 | 34 | 0 | 6 | 28 |

## Table S3. Pathogens utilized to prepare contrived samples.

| **Target** | **Supplier** | **Reference** | **LoD (microbiological units)** | **LoD (molecular units)** |
| --- | --- | --- | --- | --- |
| Astrovirus | ZeptoMetrix | 0810277CF | 11.7 TCID50/mL | 11726 copies/mL |
| Shiga-like toxin-producing STEC (serogroup *Escherichia coli* O157) *stx1*+ and *stx2*+ | ZeptoMetrix | 0801622 | 2281.5 CFU/mL | 1217 copies/mL |
| *Entamoeba histolytica* | ATCC | 30459 | 0.2 cells/mL | 7 copies/mL |
| *Plesiomonas shigelloides* | ZeptoMetrix | 0801899 | 2291 CFU/mL | 481 copies/mL |
| Rotavirus A | ZeptoMetrix | 0810280CF | 436.1 TCID50/mL | 5787 copies/mL |
| *Yersinia enterocolitica* | ZeptoMetrix | 0801734 | 2070 CFU/mL | 719 copies/mL |
| *Cryptosporidium* | Waterborne | P102C | n/a | 661 copies/mL |
| *Cyclospora cayetanensis* | US Clinical sample | LAC2825 | n/a | 53 copies/mL |
| Enterotoxigenic *Escherichia coli*  (ETEC) *lt/st* | ZeptoMetrix | 0801624 | 567 CFU/mL | 855 copies/mL |
| *Giardia lamblia* | ATCC | 30957 | 790 cells/mL | 11850 copies/mL |
| *Campylobacter jejuni* | ZeptoMetrix | 0801650 | 1660 CFU/mL | 14491 copies/mL |
| *Salmonella* | ZeptoMetrix | 0801437 | 4518.8 CFU/mL | 1441 copies/mL |
| Shiga-like toxin -producing *Escherichia coli* (STEC) *stx1*+ | ZeptoMetrix | 0801748 | 726.8 CFU/mL | 2012 copies/mL |
| Shiga-like toxin -producing *Escherichia coli* (STEC) *stx2*+ | SSI | 95211 | Not available. Dilution 3.16E-5 for lot number 1105 | |
| Adenovirus F40 | ZeptoMetrix | 0810085CF | 0.05 TCID50/mL | 979 copies/mL |
| *Shigella sonnei* | ATCC | 25931 | 0.2 CFU/mL | 488 copies/mL |

## Table S4. Contrived sample testing results obtained following testing on QIAstat-Dx Gastrointestinal Panel 2 and expressed as proportion of positive results with the two-sided 95% confidence limit interval.

| **Target** | **Proportion** | | **Two-sided 95% confidence limit** | |
| --- | --- | --- | --- | --- |
|  | **Fraction** | **Percentage (%)** | **Lower (%)** | **Upper (%)** |
| Adenovirus | 68/70 | 97.14 | 90.06 | 99.65 |
| Astrovirus | 67/68 | 98.53 | 92.08 | 99.96 |
| *Campylobacter* | 45/46 | 97.83 | 88.47 | 99.94 |
| *Cryptosporidium* | 58/58 | 100.00 | 93.84 | 100.00 |
| *Cyclospora* | 56/56 | 100.00 | 93.62 | 100.00 |
| Enteroinvasive *Escherichia coli* (EIEC) | 69/69 | 100.00 | 94.79 | 100.00 |
| Enterotoxigenic *Escherichia coli* (ETEC) | 43/43 | 100.00 | 91.78 | 100.00 |
| *Entamoeba histolytica* | 69/70 | 98.57 | 92.30 | 99.96 |
| *Giardia lamblia* | 56/56 | 100.00 | 93.62 | 100.00 |
| *Plesiomonas shigelloides* | 67/68 | 98.53 | 92.08 | 99.96 |
| Rotavirus | 69/70 | 98.57 | 92.30 | 99.96 |
| Shiga-like toxin *Escherichia coli* (STEC) *stx1*/stx2 | 200/200 * | 100.00 | 98.17 | 100.00 |
| *Escherichia coli* O157 | 67/69 | 97.10 | 89.92 | 99.65 |
| *Salmonella* | 33/33 | 100.00 | 89.42 | 100.00 |
| *Yersinia enterocolitica* | 68/69 | 98.55 | 92.19 | 99.96 |

* Higher number of test results are shown for STEC stx1/stx2 targets because they come from non-O157 STEC strains as well as STEC strains with serogroup O157.

# References

1. Buss SN, Leber A, Chapin K, Fey PD, Bankowski MJ, Jones MK, Rogatcheva M, Kanack KJ, Bourzac KM. 2015. Multicenter evaluation of the BioFire FilmArray gastrointestinal panel for etiologic diagnosis of infectious gastroenteritis. J Clin Microbiol. 53:915-25.
2. Caza M, Kuchinski K, Locher K, Gubbay J, Harms M, Goldfarb DM, Floyd R, Kenmuir E, Kalhor M, Charles M, Prystajecky N, Wilmer A. 2024. Investigation of suspected false positive norovirus results on a syndromic gastrointestinal multiplex molecular panel. J Clin Virol. 175:105732.
3. Matic N, Lawson T, Young M, Jang W, Bilawka J, Gowland L, Ritchie G, Leung V, Payne M, Stefanovic A, Romney MG, Lowe CF. 2024. Melting curve analysis reveals false-positive norovirus detection in a molecular syndromic panel. J Clin Virol. 173:105697.
4. Cointe A, Birgy A, Pascault A, Louillet F, Dufougeray A, Mariani-Kurkdjian P, Bonacorsi S. 2020. Be aware of Shiga-toxin 2f-producing Escherichia coli: case report and false-negative results with certain rapid molecular panels. Diagn Microbiol Infect Dis. 98:115177.
