# Supplementary figures and images for: Multicenter evaluation of the QIAstat-Dx Gastrointestinal Panel 2, a multiplex PCR platform for the diagnosis of acute gastroenteritis
Source: J Clin Microbiol. 2025 Jul 11;63(8):e01983-24. doi: 10.1128/jcm.01983-24 (PMC12345276; doi:10.1128/jcm.01983-24)

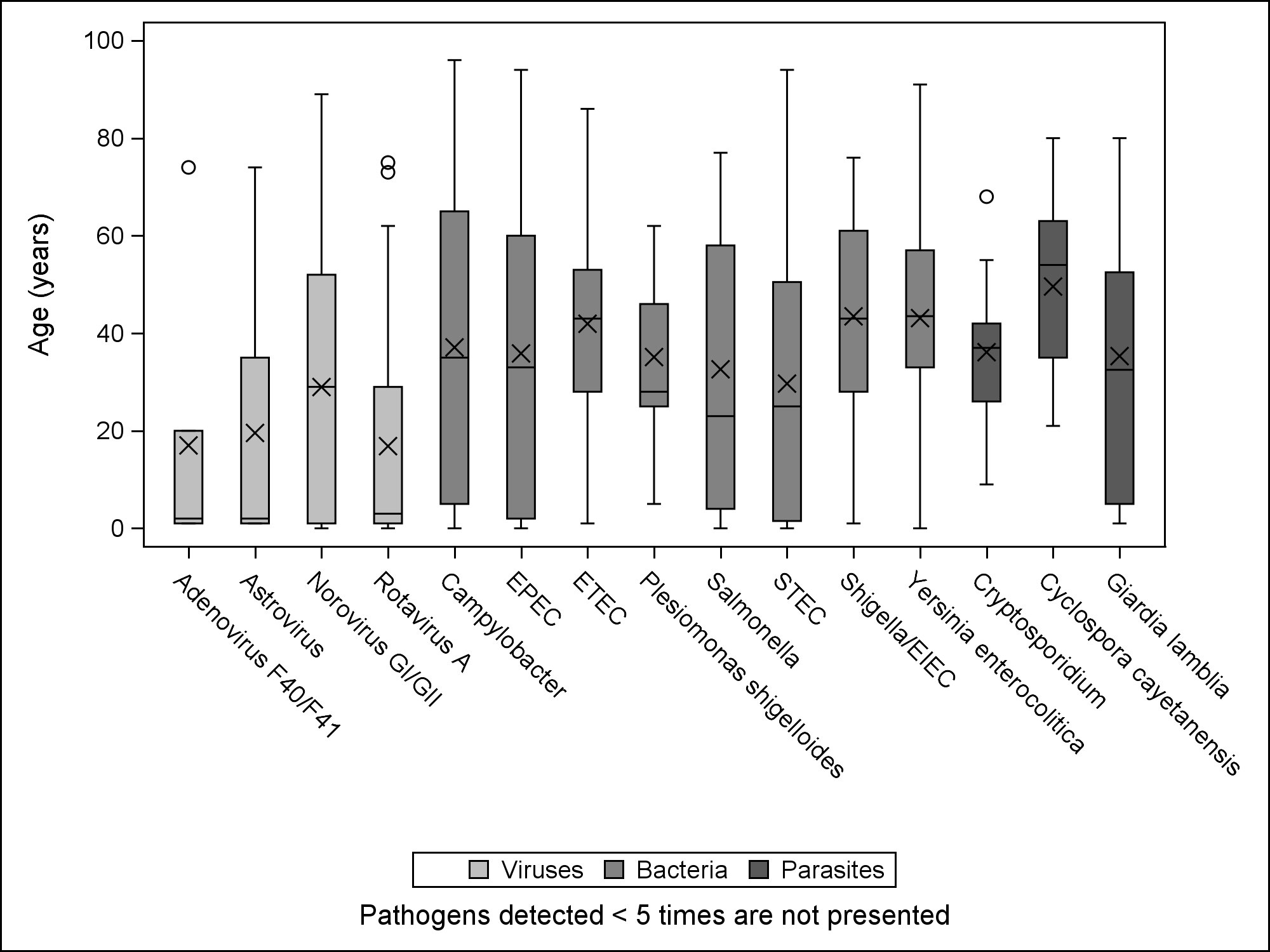

Supplement: Figure S1 — Median and quartiles of age of patients whose stool samples tested positive for various pathogens on the QIAstat-Dx Gastrointestinal Panel 2. [file jcm.01983-24-s0002.tiff]

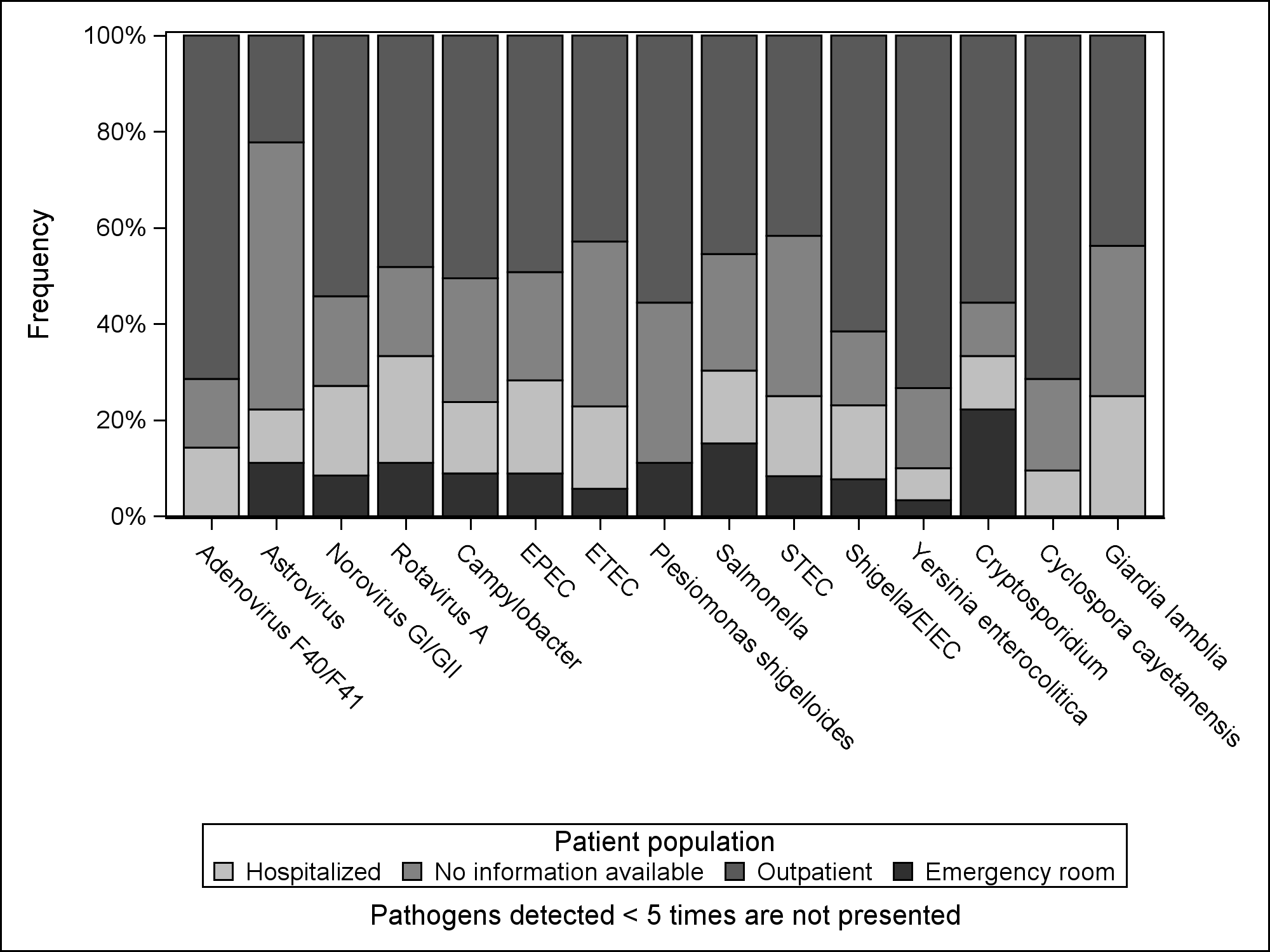

Supplement: Figure S2 — Source of the pathogens according to patients' status within the healthcare system. Pathogens detected <5 times are not presented. [file jcm.01983-24-s0003.tiff]

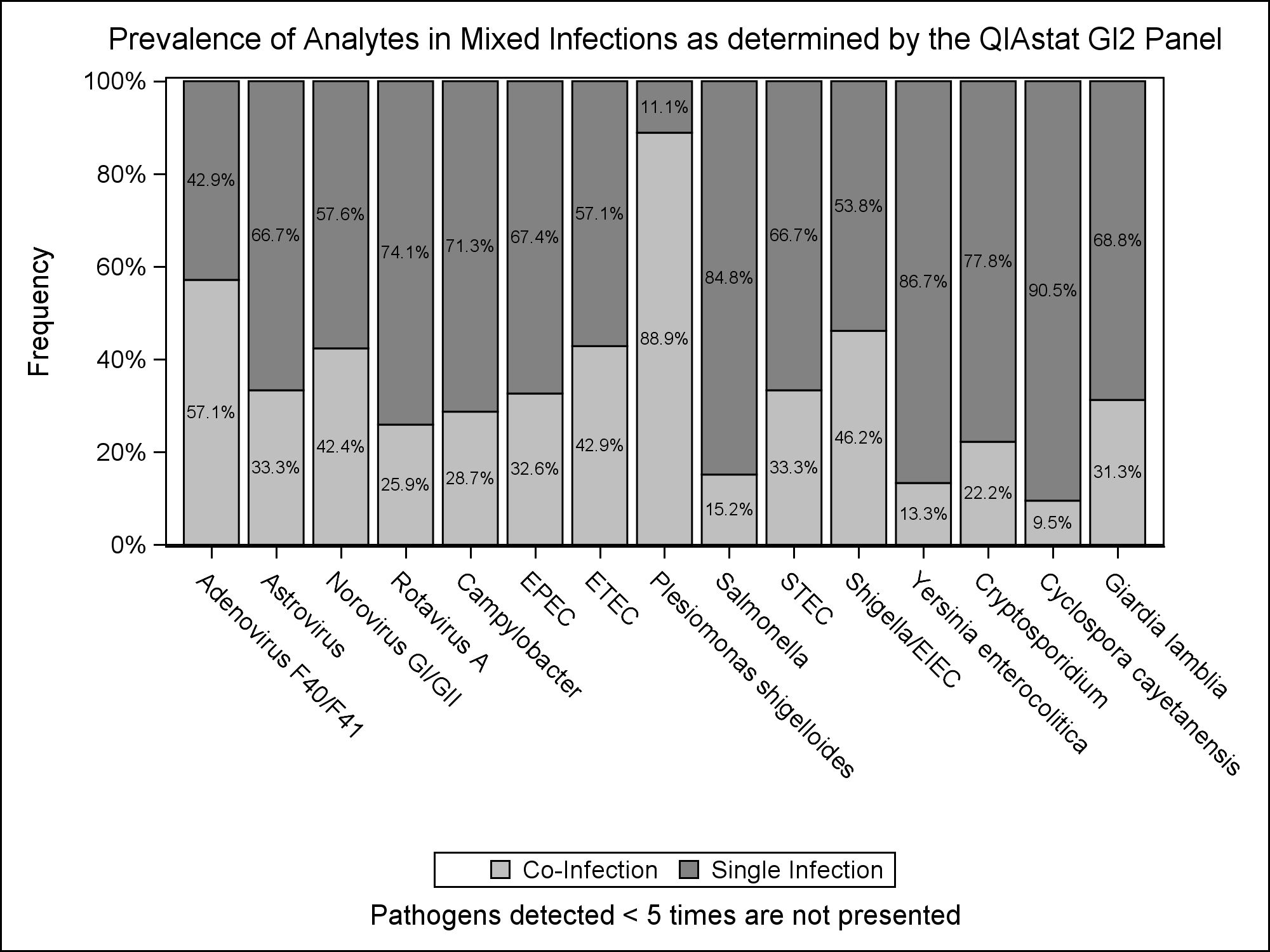

Supplement: Figure S3A — Frequency of single and co-infections for the various pathogens as detected following testing on the QIAstat-Dx Gastrointestinal Panel 2. [file jcm.01983-24-s0004.tiff]

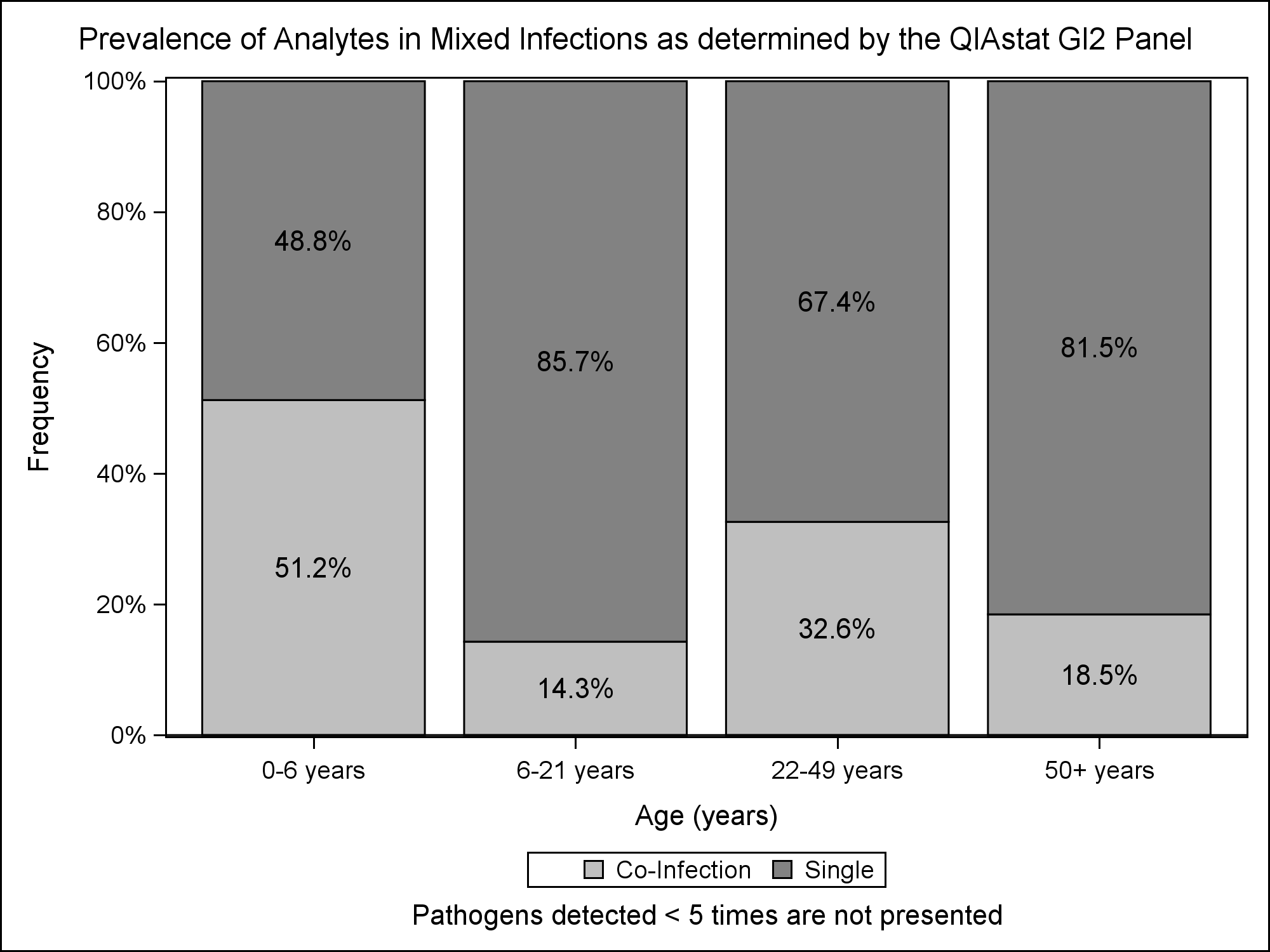

Supplement: Figure S3B — Percentages of single and co-infections within the various age groups of patients. [file jcm.01983-24-s0005.tiff]
